# Dr. Roberton in Answer to Dr. Baillie

**Published:** 1816-07

**Authors:** John Roberton

**Affiliations:** 7, Cleveland-court, St. James's-place


*For the London Medical and Physical Journal.*

### Dr. Roberton *in Answer to Dr. Baillie.*

(To Dr. Baillie.)

SIR,

I MUST trouble you with this address, in consequence of those remarks on my book which you have been induced to insert in the Medical and Physical Journal of last month; and which I am the more disposed to notice, as I have strong reason to suspect that you have been made an accessary precisely at the moment when I was proceeding to punish a newspaper libeller, for those charges which he or his friends have now prevailed on you, sir, to reiterate against me.

That, amidst the hurry of your professional duties, this interested individual has artfully worked upon your credulity, to my disadvantage, and, by the exhibition of garbled extracts from my book, has prevailed on you to adopt the line of conduct you have pursued, will not admit of a doubt. From the most cursory examination, if made with your own eyes, you would at once have seen that in the work you abuse, I have employed no new anatomical, physiological, or pathological expression; but have strictly confined myself to such terms as have been universally employed, by every medical writer, from the days of Hippocrates to the present hour.

The memory, in common with our other mental faculties, is extremely apt to become impaired; and it is not impossible that yours, sir, may have become blunted in its vigour. It is thus that you may have forgotten the minute and invaluable descriptions, given in the celebrated works of earlier writers, even of those parts and their operations to which you object.

Were I assured that it would not hurt your peculiarly delicate feelings, I could point out to you, in the works of poets, philosophers, and medical writers, of the first eminence, passages which go far beyond mine in what you term obscenity. In the works of highly respectable medical writers, I can also point out to you all my anatomical and physiological descriptions, almost word for word; for I assure you it never was either my wish or my intention to make the profession believe I had, in these, invented any thing. I need only bring to your recollection, among a host of others, the celebrated names of Morgagni, to whose works you are indebted for so great a part of your book on Morbid Anatomy, as well as those of Sommering, Sabatier, Bichat, and, though last not least, of Dr. Baillie himself. You must also be aware that, in no course of anatomical, physiological, or obstetrical lectures, can any of the expressions I have used be possibly omitted. This every one knows, for these works are in the hands of the public, and may be examined, and the lectures are every where open for the seekers of such information. At such lectures, the teacher finds it absolutely necessary that the most secret and delicate parts of the human body should be handed about, to be subjected to the most minute inspection, in order that the most perfect knowledge of their structure and operations may be obtained. You must also be well aware that I have introduced neither name nor operation, to which analogous ones are not to be found in Linnæus's arrangement of botanical plants, so often the study of the most chastely virtuous of the female sex.

From the specimen with which you have favoured the profession, in the Medical and Physical Journal, I have no doubt, had you been as much Dr. Hunter's superior as you seem to consider yourself mine, the profession would never have been benefitted by the invaluable advantages which the obstetrical art has derived from the work of that illustrious author on the Gravid Uterus.

For similar reasons, the world would have been deprived of the lucubrations of Sir Everard Home on Stricture in the

Urethra,

Urethra, Rectum, &c. because many of his descriptions are the very quintessence of what you would term obscenity. And, if you look into Mr. Abernethy's Essay on Digestion, &c. you will find abundant exercise of your supererogatory delicacy.

We shall suppose the possibility of your existence, at the various periods in the history of medical literature, during which the healthy and morbid structure of certain parts of the human body were investigated and described, by plates, demonstrations, &c.; most certainly had you been the presiding judge over such meritorious exertions, the medical world must have been left in the grossest ignorance, and have sacrificed to your delicacy its most useful acquirements. But, thank God, we have almost always had men of liberal and enlightened minds to superintend and direct these useful pursuits. When Hippocrates was entreated by the mob (who seem to have had about as delicate an abomination to the extention of knowledge as Dr. B.) to cure Democritus of madness, he found him in the obscene occupation of dissecting, to discover the seat of the bile; and he most indelicately left him to the pursuit of such useful obscenity, and pronounced him the wisest man in Abdera. I, sir, do not pretend to be a Democritus—would I could pronounce Dr. Baillie an Hippocrates!

Whatever is new in my work, is the correction of errors of former authors, such as those of Sir Everard Home, Abernethy, and other great names; the introduction of new and important medicines into practice; and the successful treatment of several most distressing diseases, which our profession had hitherto deemed incurable. It is an incontrovertible fact, that those of our profession who follow the beaten track are allowed to glide smoothly and uninterruptedly along—no one opposes them, because they follow all that has either been said or done, whether that be inefficient, erroneous, or even hurtful. Different is the fate of him who ventures to deviate from such a beaten path, in reasoning and practice, both of which I have most decidedly done. His every endeavour to lessen human misery either subjects him to the secret and calumnious whispers of his professional brethren, or raises a nest of hornets about his ears; and there are always plenty, who stand high in the profession, at once ready and willing to lend their influence to oppose that success which would necessarily be the result of toilsome industry.

How, after these my endeavours to improve medical reasoning and practice, you could be prevailed on to direct your severity against my literary and professional labours, *solely*

*solely directed for the use and altogether for the perusal of the medical profession*, and, in the most unprovoked manner, suffer yourself to be put forward to accuse me of employing obscene expressions, I am at a loss to conceive.

You ought to have recollected that my work is a systematic dissertation on the subject of generation, on the diseases peculiar to the parts immediately connected in the multiplication of our species, and such treatment as is calculated to remove such diseased action, and thus restore the parts to their original condition. Had I, in such a work, omitted *any thing* connected with such a process, my efforts might have properly been styled garbled extracts, in some measure similar to those by which you formed your opinion of my labours. Had I not done what I did, Dr. Baillie would have very properly censured me;—doing what I did, I ought to have escaped his censure.

Had I preferred to treat on digestion, I should have found it absolutely necessary to commence by a description of the aliment, as it was first received into the body, till it was partly converted into blood, &c. and so on till it exhibited signs of vitality, while I should also have deemed it my duty to have given a minute account of the remainder of the aliment, which, being of no use in the animal economy, was expelled from the body by a part which I must not offend Dr. B.'s delicacy by mentioning even by its anatomical name.

In the same manner fistula in ano, and numerous other most distressing diseases, must neither be described nor operated for; the use of pessaries must be wholly banished from practice; and even Sir Everard Home's celebrated cases of Stricture in the Rectum, should have been delicately suppressed. There are, however, other reasons why Sir Everard's strictures in the rectum should not have been related: they did not in reality exist, at least not till Sir Everard caused a contraction of the rectum by cauterizing it with caustic bougies.

Instead also of directing my attention to the generative system, I had written on the Taliacotian art of putting on noses, I must have been led to make some remarks on the importance of such an art. Then I could scarcely avoid stating, that "a nose was certainly a very handsome ornament to the face of man, and that there could scarcely be a more disgusting spectacle than a face without a nose." These words are the very counterpart of those I have applied to certain other parts, in particular states of disease, which I understand from the attorney of Dr. Baillie's friend, Mr. Scott, editor of the Champion, has greatly offended the doctor's delicacy.

Fortunately

Fortunately for me, Sir, and most unfortunately for you, it is acknowledged by universal consent, that the greater part of a physician's professional duties are really what you would term obscenities. We cannot discharge these duties, with any sort of advantage to the public, without being daily compelled to make statements, to put questions, to perform duties which he would find absolutely necessary, but which you, Sir, would deem the very height of obscenity. How you, therefore, can, with a shadow of consistency, set your face against the detail in books of professional facts, in order that at least our younger professional brethren may be instructed in the secrets of their art, I dare say you will unfold to us. Even allowing you the full force of your intention, you have not ventured to object to any one of my practical doctrines, and even now I call on you either to do that, or to vindicate the conspicuously erroneous opinions of Sir Everard Home, or any other author, on whom I have animadverted for the benefit of mankind; and of course if you decline this, I must conclude you cannot; and, if you attempt to support them by argument, I pledge myself to prove you wrong.

From the editor of the Champion, or his attorney, I should not have been surprised, but I did not expect to find Dr. Baillie in such company.

Had you now addressed me privately, as it occurs to my recollection you once had the kindness to do, when in one of the letters with which (if I mistake not) you then honored me, you, if I remember rightly, apprised me that insinuations had been made to you, directly intended to injure me, I am sure I could now have satisfied you as fully as I then did, that it was no honourable or even honest informant who sought to prejudice you against me, on grounds not less false and unjustifiable than those which you have now, Sir, unhappily adopted. What I allude to, you doubtless recollect, and I believe I addressed you by letter on the subject. Your perusal of the book must have fully satisfied you how far you were misinformed when you were told I intended to publish one of your private letters; for, were I even one of those very cautious people who preserve the letters they may receive, I should not deem myself warranted to publish them on slight occasions. From the above circumstance, I think you might, without any imputation on your judgment or your morality, have still continued the same caution against such *friendly* insinuations.

Thus, Sir, after more than twenty of the most valuable years of my life have been consumed, in attempts to render the subjects in that book more scientific and more practically cally useful, I have, during a great part of that time, been the mark of a set of interested conspirators, who never dared show themselves openly, but, like assassins, struck behind another's shield at my moral and professional reputation; and who systematically, unceasingly, and unrelentingly, have harassed and persecuted me, without allowing me any sort of opportunity of stepping forward in vindication of my rights and my character.

But, Sir, the time is perhaps at no great distance when your deliberate reflexion will regret this easy compliance with my enemies. At some fortunate moment the hurry of professional duty may abate, and you may condescend to examine my anatomical, physiological, and pathological labours, not, as in the present instance, in garbled extracts, from which no man can judge aright, but by the consistency and the practical utility of the whole.

Possessing, perhaps, less vanity than falls to the common lot of man, I do not calculate on posterity's taking much trouble either about me or my exertions. If, however, (for human proceedings are greatly influenced by accident) after times shall devote any attention to these proceedings, a more awful period approaches. The machinations of my enemies, the slanders of a newspaper editor, my own labours, and even the lucubrations of Dr. Baillie, must find their level; when party feuds have ceased, and malignant spirits have slept; when newspaper interests and personal animosities have alike expired; when posterity shall judge on our characters by the result of our principles, and no name nor rank shall weigh in the balance—full, fair, and final justice shall be done upon us all. It will then be decided which among us has contributed to the alleviation of human misery; our opinions will be tried by their truth, our services by their utility, and our reputation by our services. In the certainty of such a tribunal, I console myself even in Dr. Baillie's censure; it supports me against newspaper malice and professional jealousy.

I shall close this extremely painful task by entreating you not to misconceive me. You may know, Sir, I am not altogether independent of the world; the severity of your attack on me presupposes this knowledge.; but you must not carry it farther in the supposition that I am not independent of *you;* your acceptance of my volume, with its heaviest faults upon its page, was the only favor that I ever asked or expected from you. The possession of conscious truth, the acquirement of new facts, in the course of long study, exempt me from the solicitation of Dr. Baillie's kindness to any greater extent, at least, than the common duties of life.

I disregard the intrigues of my adversaries, and I scarcely request him to forbear assisting them; though I might reasonably expect that a man, established in his profession, would not close against me the avenue through which he so successfully passed, and that he would tolerate the honest endeavors of one wishing to arrive at such eminence as his industry may entitle him.

I shall now take leave of you after recommending to your attention the following impressive words:—"Go not forth *hastily* to strive, lest thou know not what to do in the end thereof, when thy neighbour hath put thee to shame."

I have the honor to be,

Your most obedient and very humble servant,

JOHN ROBERTON.

7, *Cleveland-court, St. James's-place;*
*June* 12, 1816.

---

*For the London Medical and Physical Journal.*

